# Sigmoid Colon Metastasis from Endometrioid Carcinoma after a Long Disease-Free Interval Following Radiotherapy for Vaginal Recurrence: A Case Report and Review of the Literature

**DOI:** 10.70352/scrj.cr.26-0134

**Published:** 2026-06-25

**Authors:** Kazuki Yokoyama, Takuya Miura, Hajime Morohashi, Akiko Suto, Yuki Yoshihara, Tadashi Yoshizawa, Shintaro Goto, Daiki Kasai, Keinosuke Ishido, Kenichi Hakamada

**Affiliations:** 1 Department of Gastroenterological Surgery, Hirosaki University Graduate School of Medicine, Hirosaki, Aomori, Japan; 2 Department of Anatomic Pathology, Hirosaki University Graduate School of Medicine, Hirosaki, Aomori, Japan

**Keywords:** endometrial cancer, endometrioid carcinoma, colorectal metastasis, late recurrence, distant recurrence

## Abstract

**INTRODUCTION:**

Colorectal metastasis from endometrioid carcinoma is exceedingly uncommon and has been reported only in isolated case reports. We describe a case of sigmoid colon metastasis occurring after a long disease-free interval following radiotherapy for vaginal recurrence.

**CASE PRESENTATION:**

A 76-year-old woman presented with bloody stool. She had undergone hysterectomy for endometrioid carcinoma 9 years ago and had achieved a complete response after chemotherapy and radiotherapy for vaginal recurrence 5 years ago. Colonoscopy revealed a circumferential tumor at the sigmoid–descending colon junction, and laparoscope-assisted sigmoidectomy was performed. Postoperative pathological and immunohistochemical examinations demonstrated that the lesion represented metastatic endometrioid carcinoma. The patient subsequently received adjuvant paclitaxel plus carboplatin and has remained disease-free for 25 months.

**CONCLUSIONS:**

This case shows that distant colorectal metastasis may occur even after a long disease-free interval following treatment for endometrioid carcinoma. Careful evaluation for late recurrence remains important, and complete surgical resection may provide effective disease control when metastasis is limited and amenable to resection.

## Abbreviations

CDX2
: caudal-type homeobox 2
CK7
: cytokeratin 7
CK20
: cytokeratin 20
ER
: estrogen receptor
PAX8
: paired box gene 8

## INTRODUCTION

Endometrioid carcinoma has an approximately 8% 5-year recurrence rate, with local recurrence, including vaginal recurrence, being the most common pattern of failure.^1)^ Distant metastases commonly involve the lungs, peritoneum, liver, and bones,^2)^ whereas colorectal metastasis is exceedingly rare and has been described only in isolated case reports. We report a case of metastasis to the sigmoid colon that developed after a 5-year disease-free interval following radiotherapy for vaginal recurrence of endometrioid carcinoma.

## CASE PRESENTATION

A 76-year-old woman presented to a primary physician with a chief complaint of bloody stool. Nine years ago, she had undergone a total hysterectomy with bilateral salpingo-oophorectomy and pelvic lymphadenectomy for endometrioid carcinoma in the Department of Gynecology at our hospital. Her disease had been diagnosed as pT3aN0M0, Stage IIIA, with positive peritoneal cytology. Six years ago, she developed a vaginal recurrence and received 6 cycles of paclitaxel plus carboplatin chemotherapy, followed by external beam radiotherapy (54 Gy in 27 fractions) for the recurrent tumor, achieving a radiological complete response. A left ureteral stent had been placed for hydronephrosis associated with the vaginal recurrence, and she subsequently remained disease-free for 5 years. Her abdomen was soft and flat without tenderness, and a lower midline surgical scar was observed. Laboratory tests were almost within normal limits. Tumor markers were not elevated, with CEA 0.9 ng/mL and CA19-9 7 U/mL. CA125 measured 1 month ago during gynecologic follow-up was within the normal range (13 U/mL). Colonoscopy revealed a circumferential tumor at the sigmoid–descending colon junction (**Fig. 1**). Gastrografin enema demonstrated an apple-core sign, and the endoscope could not be advanced beyond the stenotic segment. Biopsy specimens were obtained from the lesion. Contrast-enhanced CT showed wall thickening at the same location (**Fig. 2**), without evidence of lymph node involvement or distant metastasis. Based on these findings, the patient was clinically diagnosed with primary sigmoid colon cancer and was referred to our department for surgery before the biopsy results were available. She was admitted the following day and underwent laparoscope-assisted sigmoidectomy 4 days later. Laparoscopic inspection of the abdominal cavity revealed a tumor at the sigmoid–descending colon junction. Although the lesion was adherent to the small intestine, no definitive invasion was identified. Peritoneal dissemination and endometriotic lesions were absent. No radiotherapy-related adhesions or fibrosis were observed within the radiotherapy field for the local recurrence. After adhesiolysis of the small intestine, laparoscopic sigmoidectomy with a systematic D3 lymph node dissection was completed as planned in accordance with the standard surgical strategy for primary sigmoid colon cancer. The histopathological findings of the preoperative biopsy, which had not been available at the time of surgery, became known after surgery. Biopsy revealed a tubular adenocarcinoma with relatively uniform, well-formed glands and no apparent irregular glandular architecture as seen in conventional colorectal adenocarcinoma. Immunohistochemical profiling was consistent with endometrioid carcinoma involving the colon. In the resected specimen, tumor cells morphologically similar to those identified in the biopsy were present. Their cytologic and architectural features closely resembled those of the uterine endometrioid carcinoma that had been resected 9 years ago (**Fig. 3**). The colonic tumor showed no serosal involvement (**Fig. 3A**, shaded area). Elastic Van Gieson staining demonstrated vascular invasion (**Fig. 3B**). Immunohistochemically, the tumor was positive for ER, PAX8, CK7, and vimentin, and negative for CDX2 and CK20 (**Fig. 4**). No pathological lymph node metastasis was identified in the resected regional lymph nodes. Based on these histological and immunophenotypic findings, the lesion was diagnosed as metastatic endometrioid carcinoma to the sigmoid colon. The patient had an uneventful postoperative course and was discharged on POD 8. She subsequently received 6 cycles of paclitaxel plus carboplatin as adjuvant chemotherapy in the Department of Gynecology at our institution. She remains disease-free 25 months after sigmoidectomy.

**Fig. 1:**
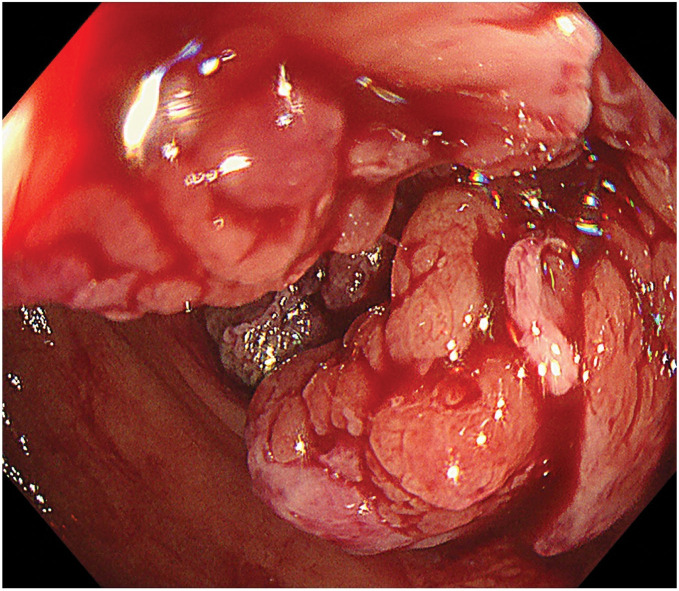
Colonoscopic findings. Colonoscopy showed a tumor occupying the lumen of the sigmoid colon.

**Fig. 2:**
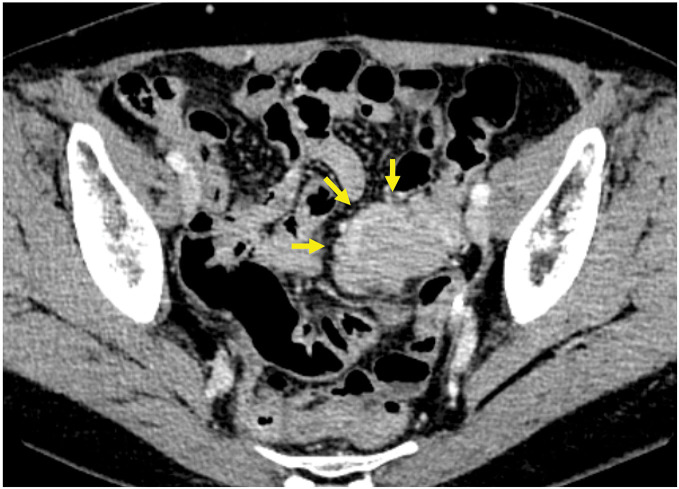
Contrast-enhanced CT. Contrast-enhanced CT showed wall thickening of the sigmoid colon due to the tumor (arrows). No adjacent organ invasion or peritumoral fat stranding was observed.

**Fig. 3:**
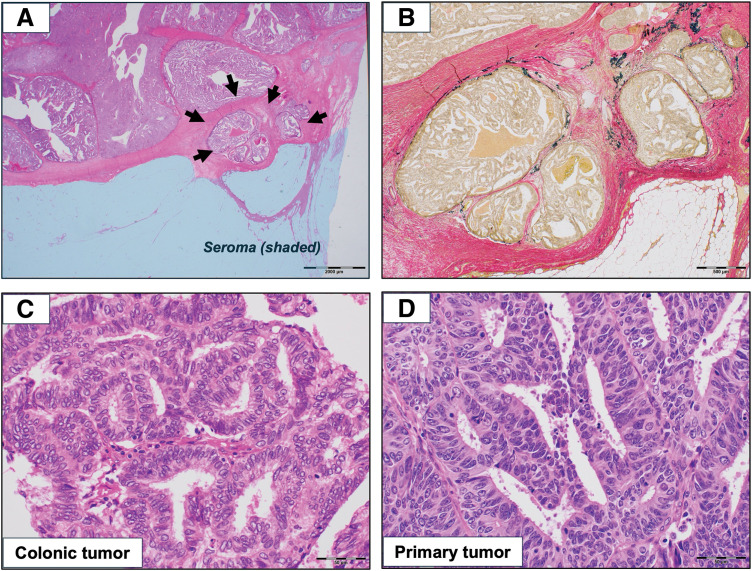
Postoperative pathological findings. (**A**) Low-magnification image of the colonic metastatic lesion showing tumor extension from the mucosa through the muscularis propria without serosal involvement (shaded area). Vascular invasion is indicated by the arrows. (**B**) Mid-magnification image of Elastica van Gieson staining of the area indicated by the arrows in (**A**), demonstrating vascular invasion. (**C**) High-magnification image of the colonic metastatic lesion. (**D**) High-magnification image of the primary uterine tumor. The colonic tumor cells showed histological similarity to the primary endometrioid carcinoma.

**Fig. 4:**
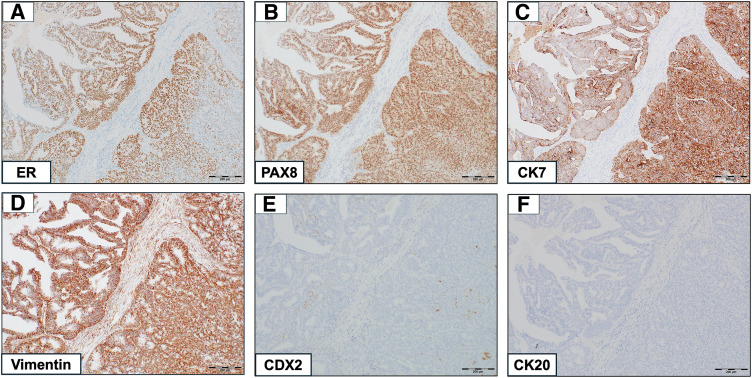
Immunohistochemical findings of the resected colonic tumor. (**A**) ER, (**B**) PAX8, (**C**) CK7, (**D**) vimentin, (**E**) CDX2, and (**F**) CK20. These immunoprofiles were diagnostic of metastatic endometrioid carcinoma.

CDX2, caudal-type homeobox 2; CK7, cytokeratin 7; CK20, cytokeratin 20; ER, estrogen receptor; PAX8, paired box gene 8

## DISCUSSION

We performed surgery for a sigmoid colon metastatic recurrence that developed 9 years after surgery for endometrioid carcinoma and 5 years after treatment for vaginal recurrence. Although direct invasion of the colon or recurrence due to peritoneal dissemination from endometrial cancer is occasionally observed, colorectal metastasis as a distant hematogenous or lymphatic spread is rare. To our knowledge, a PubMed search of all available years up to September 2025 using the terms “endometrial cancer,” “colonic metastasis,” and “colorectal metastasis,” together with screening of related articles, identified 14 cases in total.^3–16)^ No language restrictions were applied. These reported cases and our case are summarized in **Table 1**. The histological subtype was endometrioid carcinoma in 13 cases and serous carcinoma in 2 cases. Only about half of the reported cases were correctly diagnosed as colorectal metastasis before surgery, which suggests the diagnostic difficulty. The median interval from primary tumor surgery to colorectal metastasis was 6.5 years. This finding indicates that such metastases may occur as late recurrences. Muratori et al. summarized recurrences occurring more than 10 years after primary surgery for endometrial cancer and reported recurrence sites in the lung (3 cases), local or pelvic sites (3 cases), surgical scar (1 case), and colorectum (2 cases).^17)^ Although based on a limited number of cases, these findings suggest that atypical patterns of recurrence, such as colorectal metastasis, may occur in late recurrences. Including our case, there were 2 recurrent cases before the colorectal metastasis, and both were vaginal recurrences. None of the reported cases experienced recurrence after surgical resection of the colorectal metastasis during the reported follow-up period. These findings suggest that surgical resection of a colorectal metastasis may contribute to disease control.

**Table 1:** Summary of 14 previously reported cases and the present case of colorectal metastasis from endometrial cancer

No.	Author	Publication year	Age (year)	Subtype	Location	Time to colorectal metastasis after primary surgery (year)	Recurrence status before colorectal metastasis	Confirmed preoperative diagnosis	Surgery	Follow-up (month)
1	Addison et al.^3)^	2012	62	Endometrioid carcinoma	Appendix	10	−	−	Appendectomy	N/A
2	Wou et al.^4)^	2014	59	Endometrioid carcinoma	Rectum	6	−	−	Hartmann’s operation	11
3	Franchello et al.^5)^	2015	72	Endometrioid carcinoma	Rectum, liver	28	−	+	High anterior resection, partial resection of the liver	23
4	Hubers and Soni^6)^	2017	75	Endometrioid carcinoma	Sigmoid colon, jejunum	3	−	+	Low anterior resection, partial jejunal resection	6
5	Cardella et al.^7)^	2018	74	Endometrioid carcinoma	Sigmoid colon	7	Local recurrence	-	High anterior resection	8
6	Jauregui et al.^8)^	2021	89	Endometrioid carcinoma	Sigmoid colon	0.1	−	+	No surgical treatment	N/A
7	Koury et al.^9)^	2021	67	Endometrioid carcinoma	Sigmoid colon	N/A	−	+	Sigmoidectomy	12
8	Li and Zheng^10)^	2023	68	Endometrioid carcinoma	Rectum	5	–	+	Low anterior resection	6
9	Matías-García et al.^11)^	2023	77	Endometrioid carcinoma	Sigmoid colon	9	–	+	Sigmoidectomy	18
10	Cao et al.^12)^	2023	68	Endometrioid carcinoma	Transverse colon	2	–	+	N/A	N/A
11	Okano et al.^13)^	2023	80	Serous carcinoma	Sigmoid colon	9	–	–	Sigmoidectomy	6
12	Foong et al.^14)^	2024	62	Endometrioid carcinoma	Cecum	5	–	–	Right hemicolectomy	N/A
13	Shimura et al.^15)^	2024	79	Serous carcinoma	Sigmoid colon, peritoneum	5	–	–	Left hemicolectomy, excision of peritoneal metastases	6
14	Mukohyama et. al^16)^	2025	76	Endometrioid carcinoma	Sigmoid colon, pelvis	11	–	–	High anterior resection, pelvic tumor resection, partial jejunal resection	9
15	Our case	2026	76	Endometrioid carcinoma	Sigmoid colon	9	Local recurrence	–	Sigmoidectomy	25

N/A, not available

Immunohistochemistry is useful for differentiating primary colorectal cancer from colorectal metastasis of endometrioid carcinoma. Colorectal cancer typically shows CDX2 and CK20 positivity, whereas endometrioid carcinoma expresses ER, vimentin, CK7, and PAX8.^18)^ The immunoprofile of the tumor was consistent with the latter and lacked CDX2 and CK20. In combination with the histopathological findings, this supported a diagnosis of colorectal metastasis from endometrioid carcinoma rather than primary colorectal cancer. In the present case, the tumor showed no serosal exposure and demonstrated a growth pattern extending from the muscularis propria toward the mucosal surface. This morphology suggested that direct invasion from intra-abdominal local recurrence or disseminated lesions was unlikely. Because tumor cells were observed within vascular spaces and no pathological lymph node metastasis was identified, these findings suggested a possible hematogenous metastatic recurrence. Intraoperative peritoneal cytology should also have been performed at the time of sigmoidectomy to help assess the route of recurrence. Other reports have described endometrioid carcinoma arising in the colon in association with ectopic endometriosis.^19,20)^ However, in the present case, the patient had no history of endometriosis, and no evidence of endometriotic lesions was identified in the abdominal cavity and resected specimen, making this mechanism improbable. Chronic radiation-induced injury to the intestine may cause a variety of complications, including fibrosis, stricture, fistula formation, and adhesion.^21)^ In this case, radiotherapy had been performed as localized treatment for vaginal recurrence, so its effect on the operative field was expected to be limited. We began with laparoscopic inspection through the umbilical port and planned conversion to open surgery if severe adhesions were encountered. However, no apparent radiation-induced changes were observed, and laparoscopic surgery was completed safely.

Radiotherapy for vaginal recurrence has been reported to achieve a 5-year pelvic control rate of 82%–92% and a 5-year overall survival rate of 50%–83%^22–26)^ and is therefore regarded as one of the standard treatment options. However, it has also been reported that approximately one-fourth of patients develop distant progression after salvage radiotherapy (5-year metastasis-free interval of 75.5%),^23)^ indicating the need for appropriate post-treatment management. Because distant metastases are associated with poor prognosis in endometrial cancer,^26)^ controlling metastatic lesions that occur after radiotherapy is a critical issue. Complete resection is an important prognostic factor for distant metastatic recurrence; therefore, surgery should be considered when metastases are resectable.^27,28)^ Although colorectal metastasis is rare, surgical treatment can still offer effective disease control when complete resection is achievable, as in the present case.

In this case, no definitive pathological diagnosis could be made before surgery. Based on the endoscopic appearance, the lesion was regarded as a primary sigmoid colon cancer, and surgery was performed. A preoperative diagnosis of colorectal metastasis from endometrioid carcinoma would have been ideal. However, definitive diagnosis would have required additional immunohistochemical evaluation, and early surgical intervention was considered appropriate because the lesion exhibited stenosis. If metastatic endometrioid carcinoma had been diagnosed preoperatively, we would have performed PET-CT to search for other metastatic lesions and discussed the treatment strategy with the Department of Gynecology. We would also have adopted a more careful surgical approach, including peritoneal cytology, before proceeding to sigmoidectomy. Even after a prolonged disease-free interval, late recurrence of endometrial cancer should be kept in mind. Although such recurrence patterns are rare in long-term survivors after treatment for endometrial cancer, we should consider this possibility when determining the treatment strategy.

## CONCLUSIONS

We encountered an extremely rare case of colorectal metastasis occurring 9 years after primary surgery and 5 years after radiotherapy for vaginal recurrence of endometrioid carcinoma. This case suggests that colonic metastatic recurrence, although rare, should be considered in long-term survivors of endometrial cancer. These findings illustrate the importance of recognizing atypical late recurrence when planning surgery in long-term survivors of endometrial cancer and suggest that complete surgical resection may provide effective disease control when colorectal metastasis is amenable to resection.

## DECLARATIONS

### Availability of data and materials

Data sharing is not applicable to this case report, as no new datasets were generated or analyzed.
